# Effects of Escitalopram on Endoplasmic Reticulum Stress and Oxidative Stress Induced by Tunicamycin

**DOI:** 10.3389/fnins.2021.737509

**Published:** 2021-10-25

**Authors:** Lixia Yang, ZhengHong Chen, Jie Li, PengJin Ding, Yiming Wang

**Affiliations:** ^1^Affiliated Hospital of Guizhou Medical University, Guiyang, China; ^2^The First Affiliated Hospital, Guizhou University of Traditional Chinese Medicine, Guiyang, China; ^3^Department of Psychiatry and Mental Health, Guizhou Medical University, Guiyang, China

**Keywords:** escitalopram, endoplasmic reticulum stress, oxidative stress, tunicamycin, major depressive disorder

## Abstract

**Background:** Major depressive disorder (MDD) was reported to be associated with endoplasmic reticulum stress (ERS) combined with oxidative stress (OS) (ERS/OS). Here, we aimed to investigate the effects of escitalopram (ESC) on blood-brain barrier (BBB) permeability and ERS/OS-related pathways in brain microvascular endothelial cells (bEnd.3 cells) induced by tunicamycin (TM).

**Methods:** bEnd.3 cells were divided into four groups: control, TM, ESC, and ESC + TM groups. CCK-8 and flow cytometry were used to detect cell survival and apoptosis, respectively. The expression levels of proteins involved in cell permeability and ERS/OS-related pathways were assessed by western blot and immunofluorescence. Malondialdehyde (MDA) concentration and superoxide dismutase (SOD) activity were determined by commercial kits.

**Results:** We revealed that TM-induced bEnd.3 cells exhibited remarkably decreased viability and increased apoptosis rate, while ESC treatment reversed these changes. Additionally, TM treatment resulted in markedly increased PERK, GRP78, ATF6, XBP1, and CHOP protein expression levels. On the contrary, the expression of PERK, GRP78, XBP1, and CHOP was obviously reduced in TM-induced bEnd.3 cells after ESC treatment. Moreover, TM significantly reduced the expression of p-eNOS and P-gp and increased the expression of CaMKII and MMP9 compared with the control group. However, ESC reversed these changes in TM-induced bEnd.3 cells. Furthermore, the expression of SOD was significantly decreased, while MDA was significantly increased by TM treatment. In contrast, the expression of SOD was dramatically increased, while MDA was remarkably decreased by ESC treatment.

**Conclusion:** Our results demonstrated that ESC can inhibit ERS/OS and BBB permeability of TM-induced bEnd.3 cells. ESC may alleviate cognitive impairment and prevent comorbidities in MDD patients through ERS/OS.

## Introduction

Major depressive disorder (MDD) is a prevalent disorder, clinically characterized by persistent low mood, loss of interest, tardiness of thinking and reduced activity (Vance and Winther, 2020). MDD seriously affect the patient’s work life and social inter-human relations. The pathogenesis of depression is complex, and many hypotheses are involved in disorders of monoamine neurotransmitters such as 5-HT, immunity, oxidative stress, and cell apoptosis, inflammatory response (Diniz et al., 2018; Lin et al., 2018). The current treatment mainly includes tricyclic antidepressants, serotonin and norepinephrine reuptake inhibitors, and selective serotonin reuptake inhibitors (Ferrari and Villa, 2017). However, these medicines have limited effects, and only a third of the patients have an improvement in, and more than 30% patients have persistent symptoms (Mahableshwarkar et al., 2015). Therefore, understanding the molecular mechanisms of the pathophysiology of MDD is of great importance for finding better strategies.

Endoplasmic reticulum stress (ERS) is a protective response of cells. A certain degree of ERS can activate the expression of protective molecules such as endoplasmic reticulum molecular chaperones to resist stress and maintain survival. ERS was reported to play a key role in a variety of cell signaling processes such as apoptosis and inflammation (Mao et al., 2020). ERS has received extensive attention in the study of the mechanism of neuronal apoptosis after cerebral ischemia, and its molecular markers include UPR, ATF, PERK, GRP78, and Caspase-12 (Yi et al., 2021). A previous study uncovered that ER stress response pathway is activated in the pathogenesis of depression disorder, suggesting that ER stress stimulation is a new target for the treatment of depression (Nevell et al., 2014). In addition, Oxidative stress (OS) and the decreased function of endogenous antioxidant substances such as superoxide dismutase (SOD) play an important part in the pathogenesis of depression (Galecki et al., 2009). Some scholars have hypothesized that ERS/OS may be a new pathogenetic mode of MDD, and these two kinds of stress and their downstream signaling pathways are expected to be the targets of new therapies (Cao and Kaufman, 2014).

Escitalopram (ESC) is a highly selective serotonin reuptake inhibitor. Previous studies have found that ESC plays a positive neuroprotective role in several ischemic and degenerative central nervous system diseases, which not only improves depressive symptoms, but also has a positive effect on cognitive impairment (Ma et al., 2015). ESC can also improve spatial learning and memory abilities of Alzheimer’s disease patients and depressed rats by promoting synaptic plasticity in the hippocampus and reducing tau phosphorylation level (Montgomery et al., 2001; Ren et al., 2015). We previously found that ESC can inhibit the hippocampal ESR/OS response in chronic unpredictable mild stress-induced rats, reduce the apoptosis of hippocampal cells, and improve the depressive behavior of rats (Yang et al., 2018). However, it remains unclear about the mechanism underlying the regulation of ESC in ESR/OS.

In this study, we investigated the effect of ESC on viability, apoptosis, ERS, and OS of bEnd.3 cells and explored the potential mechanisms by which ESC affects ERS/OS. This study would provide a profound understanding for the mechanism underlying the effect of ESC on ERS/OS.

## Materials and Methods

### Cell Culture and Treatment

bEnd.3 cells were purchased from American Type Culture Collection (Maryland, United States). bEnd.3 cells were cultured at 37°C in an incubator of 5% CO_2_ and 95% air in dulbecco’s modified eagle medium (DMEM) containing 10% fetal serum (Solarbio, Beijing, China), 100 U/mL penicillin and 100 U/mL streptomycin (Solarbio, Beijing, China). bEnd.3 cells were divided into four groups: control group, tunicamycin (TM; Sigma, CA, United States) group (5 μg/ml TM), escitalopram (ESC; Sigma, CA, United States) group (20 μM ESC), and ESC + TM group (20 μM ESC + 5 μg/ml TM).

### Cell Counting Kit-8 Assay

The viability of bEnd.3 cells was evaluated by detecting cell proliferation via CCK-8 assay. Cells were seeded in 96-well plates with 1 × 10^4^ cells/ml. CCK-8 solution (10 μL) was added to each well and cells were cultured for 2 h. The OD 450 was measured by using a spectrophotometer (Thermo Fisher Scientific, MA, United States). The experiment had 3 replications. Cell viability (%) = (A_*treatment*_ – A_*blank*_)/(A_*control*_ – A_*blank*_) × 100%.

### Flow Cytometry

Apoptosis of bEnd.3 cells was measured via flow cytometry. Briefly, after specific treatment, cells were pelleted and washed with PBS twice. The cells were resuspended in 200 μL binding buffer and the cell number was adjusted to 1 × 10^6^/ml. The cell suspensions were treated with 5 μL Annexin V-fluorescein isothiocyanate (FITC) and incubated for 10 min in the dark at the room temperature. Then the cell suspensions were treated with 5 μL propidium iodide and incubated for 10 min in the dark at the room temperature. Apoptotic cells were analyzed using a BD FACSCalibur flow cytometer (Becton & Dickinson Company, Franklin Lakes, NJ), and data were quantified using FlowJo 10.2 software. All experiments were performed three times.

### Western Blot

The cells were lysed with RIPA buffer (Thermo Fisher Scientific, United States). A BCA protein assay kit (Thermo Fisher Scientific, United States) was used to detect the concentration of total proteins. Equal protein (50 μg) was loaded on a 10% SDS gel and transferred to polyvinylidene fluoride membranes. Subsequently, membranes were blocked in 5% milk in TBST for 2 h. Primary antibodies against PERK (1:2,000; Genetex, United States), XBP1 (1:2,000; Sigma, CA, United States), ATF6 (1:2,000; Sigma, CA, United States), CHOP (1:2,000; Sigma, CA, United States), Caspase-12 (1:2,000; Sigma, CA, United States), JNK (1:2,000; Sigma, CA, United States), GRP78 (1:5,000; Sigma, CA, United States), p-eNOS (1:5,000; Cell Signaling, MA, United States), CaMKII (1:5,000; Genetex, United States), P-gp (1:5,000; Sigma, CA, United States), MMP9 (1:5,000; Sigma, CA, United States), and β-actin (1:5,000; Abcam, MA, United States) were utilized to incubate membranes at 4°C overnight. Membranes were washed and incubated with goat anti-rabbit IgG horseradish peroxidase-conjugated secondary antibody (1:5,000; Proteintech, China) for 2 h. Western blot bands were analyzed by Bio-rad Image Lab (v5.2.1).

### Immunofluorescence

bEnd.3 cells were fixed in 4% paraformaldehyde for 15 min and then incubated in 5% bovine serum albumin with 0.1% Triton X-100 for 1 h to permeabilize the cells and block non-specific protein-protein interactions. The cells were then incubated with the anti-GRP78 (1:1,000; Sigma, United States) overnight at 4°C. Alexa Fluor 594-conjugated goat anti-rabbit IgG polyclonal antibody (1:200, Cell Signaling, MA, United States) was used as the secondary antibody. 4′,6-diamidino-2-phenylindole (DAPI) was used for nuclear staining. Immunofluorescence results were visualized by using a Zeiss Imager Z2 microscope (Carl Zeiss, Oberkochen, Germany). The average fluorescence intensity was analyzed using Image J (v1.8.0.112).

### Measurement of Ca^2+^ and NO

Fluo-3AM and DAF-FM DA dye staining Was used to detect the levels of Ca^2+^ and NO From treated bEnd.3 cells, respectively. in brief, bEnd.3 cells Were incubated in 5 μM Fluo-3AM (1:1,000; Beyotime, China) and 5 μM DAF-FM DA (1:1,000; Beyotime, China) for 30 min at 37°C. Then the cells Were wash three times With serum-free medium. Hoechst Was added to cell suspensions and cells Were incubated for 20 min at 37°C. Subsequently, cells Were washed three times With PBS. DAPI Was used for nuclear staining. Images Were observed using fluorescence microscopy (Tokyo, Japan).

### Detection of Malondialdehyde and Superoxide Dismutase Activity

The activities of MDA and SOD of bEnd.3 cells were detected using the MDA Assay Kit (Nanjing Biological Technology, Nanjing, China) and SOD Activity Detection Kit (Nanjing Biological Technology, Nanjing, China). The cells were centrifuged at 12,000 rpm for 10 min at 4°C. The supernatant was collected for the following analysis. Protein concentration was determined by BCA method. The absorbance of MDA and SOD was detected at 450 nm by the Microplate Reader (Thermo Fisher Scientific, NY, United States).

### Statistical Analysis

Data were analyzed by SPSS 19.0 and expressed as mean ± standard deviation (SD). The *t*-test was used to analyze the difference between two groups, and one-way analysis of variance (ANOVA) was selected to determine the statistical significance of more than two groups. Statistically significant differences of *P* < 0.05, and *P* < 0.01 are noted with ^∗^ and ^∗∗^, respectively.

## Results

### Effect of Escitalopram on Viability and Apoptosis of bEnd.3 Cells

**FIGURE 1:**
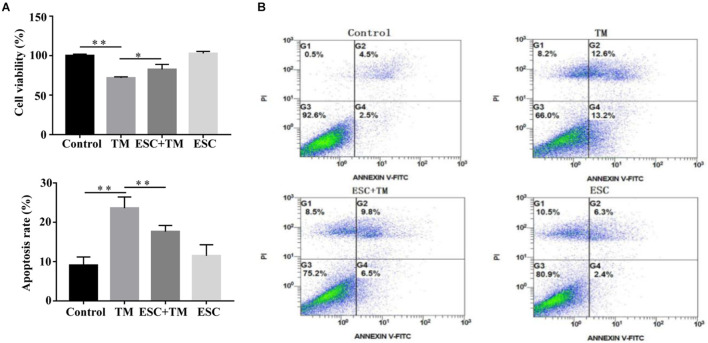
Escitalopram (ESC) enhanced viability of bEnd.3 cells induced by tunicamycin (TM). **(A)** The effect of ESC on viability of bEnd.3 cells. Cell viability assays of bEnd.3 cells were measured by CCK8. **(B)** The effect of ESC on apoptosis of bEnd.3 cells. Apoptosis levels of bEnd.3 cells was analyzed by Annexin V-FITC/PI double-staining. Data are presented as mean ± SD. *p < 0.05. **p < 0.01.

In order to evaluate the effect of ESC on viability and apoptosis of bEnd.3 cells, CCK8 assay and flow cytometry were carried out. As shown in Figure 1A, TM significantly decreased bEnd.3 cells viability compared with the control group, while ESC + TM treatment remarkably increased bEnd.3 cells viability compared with TM treatment. In addition, apoptosis of bEnd.3 cells stimulated by TM was obviously increased compared the control group (Figure 1B). However, ESC dramatically reduced the apoptosis of bEnd.3 cells induced by TM.

### Effect of Escitalopram on Endoplasmic Reticulum Stress-Related Pathways in bEnd.3 Cells

**FIGURE 2:**
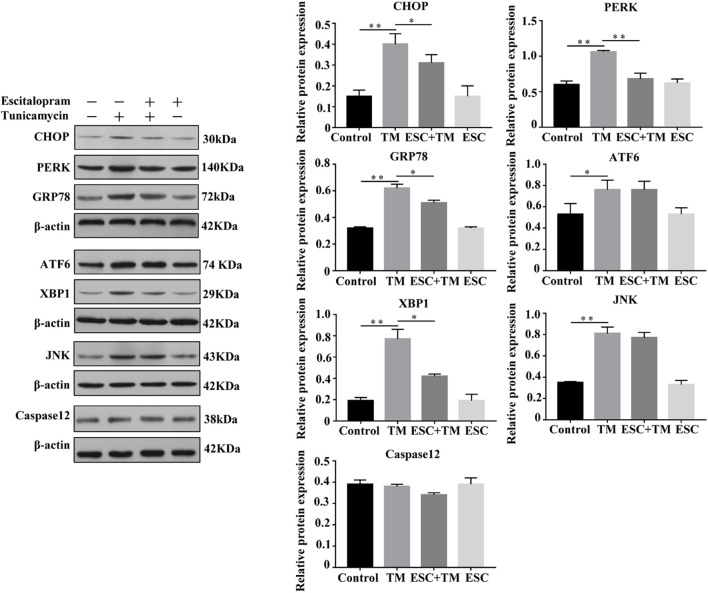
Effect of escitalopram (ESC) on ERS-related pathways of bEnd.3 cells. Western blot analysis of CHOP, PERK, GRP78, ATF6, XBP1, JNK, and Caspase12 protein after tunicamycin (TM) and ESC treatment in bEnd.3 cells. β-actin served as control. Data are presented as mean ± SD. *p < 0.05. **p < 0.01.

**FIGURE 3:**
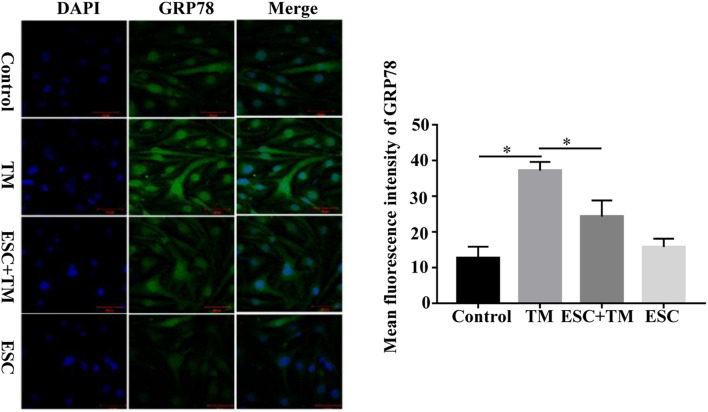
Effect of escitalopram (ESC) on GRP78 expression in bEnd.3 cells. GRP78 expression was measured by immunofluorescence microscopy (400×). The mean fluorescence intensity was computed by using ImageJ (v1.8.0.112). Results were shown as the mean ± SD. *p < 0.05.

In order to evaluate the effect of ESC on ERS-related pathways in bEnd.3 cells, the expressions of five representative pathways of ERS, PERK-CHOP pathway, ATF6-CHOP pathway, XBP1 pathway, JNK pathway, and Caspase12 pathway were evaluated. Firstly, we examined the effect of ESC on GRP78 and CHOP protein expression, two ERS markers. As shown in Figure 2, TM obviously increased the protein levels of GRP78 and CHOP in bEnd.3 cells. However, ESC + TM remarkably suppressed the protein levels of GRP78 and CHOP compared with the TM group. In agreement with the WB result, the immunofluorescence test showed that the fluorescent expression of GRP78 in the TM group was obviously higher than that in the control group (Figure 3). However, the ESC + TM group showed a prominent decrease in the fluorescent expression of GRP78 compared with the TM group. These results indicated that ESC can inhibit TM-activated ERS. Next, the protein expression of PERK, ATF6, XBP1, JNK, and Caspase12 was measured. WB analysis demonstrated that PERK, ATF6, XBP1, and JNK protein expression was significantly upregulated in the TM group as compared with the control group, while ESC + TM remarkably decreased the expression of PERK and XBP1 as compared with the TM group (Figure 2). There was no significant difference regarding ATF6 and JNK protein expression between the TM and ESC + TM groups. There were no significant changes in the protein expression of Caspase12 among all groups.

### Effect of Escitalopram on MMP9, eNOS Uncoupling and Ca^2+^/CaMKII in bEnd.3 Cells

**FIGURE 4:**
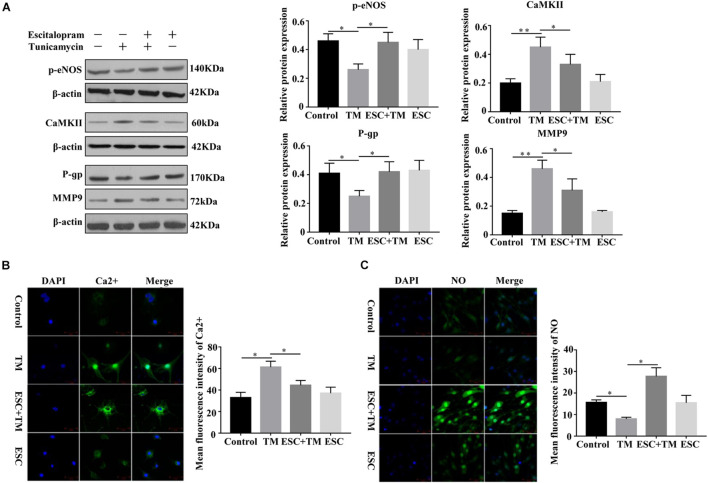
Effect of escitalopram (ESC) on the expression of MMP9, eNOS uncoupling and Ca^2+^/CaMKII in bEnd.3 cells. **(A)** Western blot analysis of p-eNOS, CaMKII, P-gp and MMP9 expression after tunicamycin (TM) and ESC treatment in bEnd.3 cells. β-actin served as control. **(B)** Effect of ESC on the level of Ca^2+^ in bEnd.3 cells. The level of Ca^2+^ was determined using Fluo-3AM fluorescent probe under a fluorescence microscopy (400×). **(C)** Effect of ESC on the level of NO in bEnd.3 cells. The production of NO was measured using DAF-FM DA fluorescent probe (400×). The mean fluorescence intensity was computed by using ImageJ (v1.8.0.112). *p < 0.05. **p < 0.01. Results were shown as the mean ± SD.

In order to investigate the role and mechanism of ESC in blood-brain barrier (BBB) permeability of bEnd.3 cells, we detected MMP9, P-gp, CaMKII, and p-eNOS protein expression by WB, and detected Ca^2+^ and NO expression by Fluo-3AM and DAF-FM DA staining, respectively. Compared with the control group, the protein expression of CaMKII and MMP9 was increased, while the protein expression of p-eNOS and P-gp protein was decreased in the TM group (Figure 4A). On the contrary, in comparison with the TM group, the protein expression of CaMKII and MMP9 was decreased, while the p-eNOS and P-gp protein expression was increased in the ESC + TM group. In addition, the mean fluorescence intensity of Ca^2+^ significantly increased, while the mean fluorescence intensity of NO obviously decreased in the TM group compared with the control group (Figures 4B,C). In contrast, compared with the TM group, the mean fluorescence intensity of Ca^2+^ significantly decreased, while the mean fluorescence intensity of NO markedly increased in the ESC + TM group. The results indicated that ESC could inhibit Ca^2+^ level, while promote NO level in TM-induced bEnd.3 cells.

### Effect of Escitalopram on Oxidative Stress in bEnd.3 Cells

**FIGURE 5:**
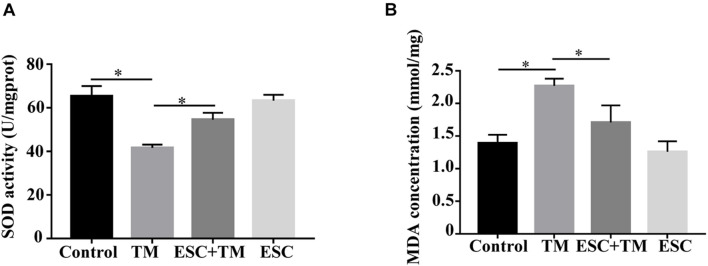
Effect of escitalopram on oxidative stress of bEnd.3 cells. **(A)** Superoxide dismutase (SOD) activity. **(B)** Malondialdehyde (MDA) concentration. ^∗^*p* < 0.05. Results were shown as the mean ± SD.

We assessed the effect of ESC on oxidative stress in bEnd.3 cells. As shown in Figure 5, TM significantly decreased the expression level of SOD and increased the expression level of MDA in bEnd.3 cells compared with the control group. In addition, their expressions were reversed by ESC treatment. These results indicate that ESC alleviate oxidative stress of TM-induced bEnd.3 cells.

## Discussion

ERS has been reported to cause cell apoptosis (Lin et al., 2012). In our study, we also found TM-induced ERS triggered apoptosis in bEnd.3 cells. There are several potential mechanisms responsible for the process of ERS-induced apoptosis. First, in this study, we found that PERK-CHOP pathway was activated in in TM-induced bEnd.3 cells, as demonstrated by increased PERK and CHOP protein expression. PERK-CHOP pathway is a main pathway that mediates ERS and drives apoptosis. PERK is an ER transmembrane sensor and exist in the ER lumen. When sustained and severe ERS occurs, the activated PERK induces CHOP expression, which initiates the CHOP-mediated apoptotic pathway, thus leading to apoptosis (Nishitoh, 2012). A previous study discovered that heat-labile enterotoxin could cause intestinal epithelial cell apoptosis via PERK-CHOP pathway (Lu et al., 2017). Another study found that reticulocalbin-1 knockdown activated ERS through the activated PERK-CHOP signaling pathway, thus leading to nasopharyngeal carcinoma cell apoptosis (Huang et al., 2020). Based on our results, we speculated that TM may cause ERS-induced apoptosis via PERK-CHOP pathway. Secondly, in our study, TM-induced bEnd.3 cells showed remarkable increased XBP1 protein expression as compared with the control group. XBP1 pathway has been reported to participate in cell survival, apoptosis, and inflammation (Yap et al., 2020). XBP1 can enter the nucleus and regulate ERS at the transcriptional level, enhance the folding ability of endoplasmic reticulum proteins, thus enhancing cell survival. However, overactive XBP1 pathway may promote apoptosis of vascular endothelial cells and neurons (Zhao et al., 2016). Wang C. et al. (2018) discovered that estrogen receptor antagonist fulvestrant promoted apoptosis of prolactinoma cells through XBP1 pathway. Therefore, XBP1 pathway may promote ERS-induced apoptosis of TM-induced bEnd.3 cells. Finally, ATF6 is a key transcriptional activator during ERS (Niu et al., 2018). The expression of the CHOP protein is under the control of ATF6 during ERS (Yu et al., 2020). ERp29 deficiency was found to have effects on sensitivity to apoptosis through ATF6-CHOP pathway during ERS (Hirsch et al., 2014). Melatonin protects against neuronal apoptosis via suppression of the ATF6-CHOP pathway in a rat model of intracerebral hemorrhage (Xu et al., 2018). The results of this study showed that the expression of ATF6 increased in TM-induce bEnd.3 cells, suggesting that the ATF6-CHOP pathway might be activated.

There is evidence that BBB permeability plays a key role in the development of MDD (Huang and Tan, 2021). MMP9 can damage the tight connections between endothelial cells, degrade the extracavascular basement membrane directly, thus increasing the permeability of BBB (Gorkiewicz et al., 2015). The expression level of serum MMP9 was increased in MDD patients and correlated with MDD severity (Domenici et al., 2010). It was also found that changes in BBB permeability were in accordance with the increased level of MMP9 in traumatic brain injury and stroke (Song et al., 2018). Pgp is an important component of BBB. The expression of Pgp protein in the brain microvascular endothelial cells of MDD patients and depressed rats was down-regulated, which promoted the permeability of BBB to neurotoxic substances (Hawkins et al., 2010). In this study, TM-induced bEnd.3 cells showed significant increased apoptosis rate and decreased survival rate after 24 h, and MMP9 expression was upregulated and Pgp expression was down-regulated, which indicated that this bEnd.3 cell model exhibit dysfunction of high cell permeability. However, ESC remarkably reversed these changes in TM-induced bEnd.3 cells. These results indicated that ESC might inhibit BBB permeability and participate in MDD progress.

eNOS is mainly expressed in vascular endothelial cells and is the main source of endodermal NO. The maintenance of a certain level of NO and eNOS *in vivo* has a positive protective effect on cells (Luiking et al., 2012). eNOS/NO was reported to exert a vital role in maintain BBB function (Zhang et al., 2019). A previous study reported that when MDD patients develop persistent cerebral hypoperfusion, the oxidative function of mitochondria of endothelial cells can be affected, leading to the increase of ROS synthesis, which in turn leading to the decrease of endothelial eNOS and NO levels, thus weakening the protective effect of them on endothelial cells (Chen et al., 2010). TM reduced the expression and phosphorylation of eNOS and the generation of NO in the rat aortic endothelial cells, which were reversed by ticagrelor (Wang X. et al., 2018). It was reported that TM inhibited eNOS levels, phospho eNOS and NO production in cardiomyocytes, and baicalin inverted these changes and attenuated ERS-induced apoptosis (Shen et al., 2014). Similarly, in the present study, TM-induced ERS/OS resulted in the decreased expression of NO and eNOS in bEnd.3 cells, while ESC reversed these changes, indicating that ESC played a protective role through regulating eNOS/NO. In addition, the Ca^2+^ signaling pathway has been found to play a key role in BBB permeability (Ding et al., 2019). Evidence indicated that after ERS is initiated, endoplasmic reticulum Ca^2+^ leaks into the cytoplasm, and cytoplasmic Ca^2+^ overload activates intracytoplasmic CaMKII, which mobilizes Ca^2+^ to move from ER into the cytoplasm and eventually causes OS (Muneer and Shamsher Khan, 2019). Simultaneously, intracellular Ca^2+^ overload and increased CaMKII expression are necessary for ERS-induced apoptosis (Wang et al., 2017). Similarly, in this study, the Ca^2+^ and CaMKII expression was significantly increased in TM-induced bEnd.3 cells. Therefore, TM may promote BBB permeability of bEnd.3 cells via Ca^2+^/CaMKII pathway.

OS is a main factor for BBB permeability in neurological disorders (Kuriakose et al., 2019). MDA is one of the active products of OS and the change of its concentration can reflect the damage degree of free radicals to the body (Zheng et al., 2018). SOD is the main antioxidant enzyme in the body, which can remove ROS of cells and play a protective role against oxidation (Tabassum and Jeong, 2019). SOD and MDA are often used to evaluate the occurrence of OS and the effect of intervention measures, and SOD is often used to detect the neuroprotective effect of antidepressants (Khan et al., 2011). The results of this experiment showed that MDA concentration was significantly increased and SOD activity was significantly decreased in TM-induced ERS cell model, indicating that ERS-induced OS occurred in this cell model. However, SOD and MDA expression was reversed by ESC + TM treatment, suggesting that ESC can reduce OS of TM-induced bEnd.3 cells. These results indicated that ESC might inhibit BBB permeability via regulating OS, which improving MDD. Additionally, ERS and OS are closely related, and they interact and induce each other (Kunitomi et al., 2020). Studies have suggested that ERS can reduce eNOS activity and gene expression, leading to vascular endothelial dysfunction, suggesting that ERS causes vascular endothelial dysfunction through its induced OS (Lenna et al., 2010; Galán et al., 2014). Wang et al. (2019) discovered that icariin can ameliorate OS of vascular endothelial cells via suppressing ERS. These researches revealed the complex relationship among ERS, OS, and endothelial cell biology. Therefore, ERS/OS is speculated to be an important mechanism leading to changes in neurovascular and BBB permeability. Inhibition of ERS/OS may be a novel strategy for the treatment of diseases associated with BBB hyperpermeability, such as MDD.

Increasing evidences have shown that fluvoxamine, a selective serotonin reuptake inhibitor, promotes neuroprotection and ameliorates depressive behavior by inhibiting ERS-mediated apoptosis (Hosoi et al., 2012; Omi et al., 2014). Our previous animal studies have shown that the improvement of depression behavior in CUMS rats by ESC may be related to the inhibition of ERS/OS (Wan, 2016; Yang et al., 2018). The present study verified some of the results *in vitro* and found that ESC can reduce the BBB hyperpermeability induced by TM-induced ERS, which was related to the inhibition of ERS/OS. In addition, ESC treatment remarkably reduced the increased protein levels of CHOP, PERK, XBP1, and CaMKII and increased the reduced expression of p-eNOS and NO in TM-induced bEnd.3 cells. Therefore, the potential mechanisms may include inhibition of XBP1, PERK-CHOP pathways, down-regulation of CaMKII expression and inhibition of eNOS decoupling. Combined with the conclusion of previous animal experiments, it can be inferred that ESC may play a positive role in alleviating cognitive dysfunction of MDD and preventing comorbidities via ERS/OS.

## Conclusion

In conclusion, TM can induce ERS/OS in bEnd.3 cells, resulting in increased cell permeability. ESC may alleviate TM-induced bEnd.3 cell hyperpermeability disorder through inhibiting the ERS/OS response via XBP1, PERK-CHOP, and CaMKII pathways and decoupling eNOS. This study provides new insights into the potential mechanisms of MDD and offers useful reference value for expanding the use of ESC in the treatment of MDD.

## Data Availability Statement

The datasets used and/or analyzed during the current study are available from the corresponding author on reasonable request.

## Author Contributions

YW designed the experiments. LY and ZC prepared the manuscript. LY, ZC, JL, and PD performed the experiments. LY, ZC, and YW analyzed the data. All authors read and approved the final manuscript.

## Conflict of Interest

The authors declare that the research was conducted in the absence of any commercial or financial relationships that could be construed as a potential conflict of interest.

## Publisher’s Note

All claims expressed in this article are solely those of the authors and do not necessarily represent those of their affiliated organizations, or those of the publisher, the editors and the reviewers. Any product that may be evaluated in this article, or claim that may be made by its manufacturer, is not guaranteed or endorsed by the publisher.
